# An Altered Circadian Clock Coupled with a Higher Photosynthesis Efficiency Could Explain the Better Agronomic Performance of a New Coffee Clone When Compared with a Standard Variety

**DOI:** 10.3390/ijms20030736

**Published:** 2019-02-10

**Authors:** Lucile Toniutti, Jean-Christophe Breitler, Charlie Guittin, Sylvie Doulbeau, Hervé Etienne, Claudine Campa, Charles Lambot, Juan-Carlos Herrera Pinilla, Benoît Bertrand

**Affiliations:** 1 CIRAD, IPME, 34 398 Montpellier, France; Lucile@toniutti.net (L.T.); herve.etienne@ird.fr (H.E.); benoit.bertrand@cirad.fr (B.B.); 2 IRD, IPME, 34 398 Montpellier, France; charlie.g57@hotmail.fr (C.G.); sylvie.doulbeau@ird.fr (S.D.); claudine.campa@ird.fr (C.C.); 3 UMR IPME, Univ. Montpellier, IRD, CIRAD, 34 398 Montpellier, France; 4 Nestlé R&D Tours, 101 AV. G. Eiffel, Notre Dame d’Oé, BP 49716, 37097 Tours CEDEX 2, France; charles.lambot@rdto.nestle.com (C.L.); JuanCarlos.HerreraPinilla@rdto.nestle.com (J.-C.H.P.)

**Keywords:** *Coffea arabica*, chlorophyll *a* fluorescence, transcriptomic approach, plant vigor, circadian clock

## Abstract

In a context where climate change is threatening coffee productivity, the management of coffee leaf rust is a challenging issue. Major resistant genes, which have been used for many years, are systematically being overcome by pathogens. Developing healthy plants, able to defend themselves and be productive even when attacked by the pathogen, should be part of a more sustainable alternative approach. We compared one hybrid (GPFA124), selected for its good health in various environments including a reduced rust incidence, and the cv. ‘Caturra’, considered as a standard in terms of productivity and quality but highly susceptible to rust, for phenotypic variables and for the expression of genes involved in the circadian clock and in primary photosynthetic metabolism. The GPFA124 hybrid showed increased photosynthetic electron transport efficiency, better carbon partitioning, and higher chlorophyll content. A strong relationship exists between chlorophyll *a* fluorescence and the expression of genes related to the photosynthetic electron transport chain. We also showed an alteration of the amplitude of circadian clock genes in the clone. Our work also indicated that increased photosynthetic electron transport efficiency is related to the clone’s better performance. Chlorophyll *a* fluorescence measurement is a good indicator of the coffee tree’s physiological status for the breeder. We suggest a connection between the circadian clock and carbon metabolism in coffee tree.

## 1. Introduction

Since the middle of the last century, rising human population and economic development have continued to put pressure on agricultural systems to produce increased crop yields. A particular concern for maintaining production levels in the context of climate change is the management of diseases, which account for up to 25% of pre-harvest crop losses in agricultural areas managed with well-developed crop protection technologies [1,2]. More specifically, in the field of Arabica coffee cultivation, global warming and unstable climate affect not only the plant but also the behavior of coffee pests and diseases. From 2008 to 2013, more intense coffee rust epidemics than those previously observed arose in Mesoamerica, from Colombia to Mexico. Meteorological anomalies, caused by ongoing global warming, are considered one of the main factors helping the emergence of this rust epidemic, and to affect both the pathogen and the physiological status of the coffee tree [3,4,5]. Management of the disease is a challenging practical problem made worse by the fact that tropical climates are favorable to disease development. Moreover, as coffee is a perennial crop, susceptible host tissue is present over long periods [6]. Rational control based on the use of fungicides at specific disease level thresholds is effective in coffee leaf rust management [7]. However, the high cost of fungicides has made them inaccessible for many producers [8]. These products are not a sustainable solution because the intensification of agricultural practices that has been witnessed over the past four decades has seen increasing environmental and health problems [9,10].

Alternative phytosanitary protection can be put in place and relies on interactions in the ecosystem to regulate pathogens and pests, as well as the deployment of a multitude of non-chemical management practices [11,12]. Therefore, holistic plant health could be an important part of the solution if one considers that a plant can be considered healthy as long as its physiological performance, determined by its genetic potential and environmental conditions, is maintained [13]. The focus should be on developing and maintaining healthy plants, so they are more resilient and more resistant by adjusting themselves to environmental conditions. This idea would comprise the cultivation of plants making better use of their resources to address the vagaries of the environment while ensuring that productivity will be sufficiently high to meet farmers’ economic needs.

The advantages of Arabica hybrid cultivars were demonstrated, for the first time, in a Kenyan breeding program [14,15]. In Central America, Bertrand et al. (2005) assessed the heterosis of F1 hybrid clones between 22% and 47%. They concluded, in a following study, that heterosis gave hybrid clones an advantage over lines in terms of productivity without increasing outputs, but also in terms of yield stability, due to their homeostasis [16].

The molecular mechanism for heterosis—widely used in agriculture since the beginning of the last century—remains largely elusive. Recently, the altered expression of circadian clock genes while maintaining the clock period was linked to hybrid vigor. Ni et al. 2009 showed that, in *Arabidopsis* hybrids and allopolyploids, increased photosynthetic and metabolic activities are linked to altered expression of the circadian clock regulators Late Elongated Hypocotyl (LHY), Circadian Clock-Associated 1 (CCA1), Timing Of CAB 1 (TOC1), and Gigantea (GI) [17]. The circadian clock of F1 hybrids and stable allopolyploids from *Arabidopsis thaliana* and *Arabidopsis arenosa* species are deregulated compared to diploid parents, leading to higher chlorophyll and starch production and an enhanced energetic metabolism [18]. The authors demonstrated that epigenetic deregulation of circadian clock regulators, which control many genes and are involved in many biological processes, may partially explain hybrid vigor [18]. In maize, higher levels of carbon fixation and starch accumulation in hybrids are associated with altered temporal gene expression of two CCA1 homologues. In these hybrids, CCA1 proteins target thousands of output genes early in the morning, as if the hybrids wake up early to promote photosynthesis, starch metabolism, and biomass accumulation [19]. Monocots produced similar results. In super-hybrid rice, yield-related QTLs are associated with gene expression changes in the circadian clock and light signaling pathways [20]. Recently, Shen and collaborators (2015) [21] showed that three circadian clock genes, OsCCA1, OsTOC1, and OsGI, are expressed in an above high parent pattern (AHP) at the seedling stage in rice F1 hybrids, as well as downstream genes involved in the chlorophyll and starch metabolic pathways, suggesting that the circadian rhythm pathway may be also related to heterosis in monocots. It is unknown whether a similar mechanism mediates heterosis in *Coffea arabica* hybrids.

If one considers the health of a plant to be the ability to carry out its physiological functions to the best of its genetic potential, coffee hybrid clones are undoubtedly healthier than pure line varieties regardless of the environment [13,15,16]. To test this hypothesis, we compared the phenotypic performance of two genotypes: the F1 hybrid GPFA124 clone, selected for high productivity and excellent health in various environments; and the Caturra variety, considered as a standard in terms of productivity in many coffee-producing countries. Caturra and the GPFA124 clone are susceptible to coffee rust. Recently, Echeverria-Beirute et al. (2017) [22] showed, in field conditions, that a vigorous F1 hybrid clone (H3) was more resistant to rust than the standard pure line Catuai variety.

Accurate allocation of limited resources between growth and defense is critical for plant fitness. The link between vigor and photosynthesis is particularly interesting because adaptive responses to environmental stresses are often constrained by resource availability [23]. Allocation of resources to growth can reduce the investment in defense, and allocation to defense can limit growth and competitive ability against neighboring plants [24,25]. Moreover, plants must find a precise balance in their responses to stresses [23,26]. Increased photosynthetic capacities provide plants with more resources to deal with abiotic constraints and fight effectively against pests and diseases. A better understanding of the relationships between primary photosynthetic metabolism and plant health would be particularly interesting to explore, providing new avenues for the development of coffee varieties better adapted to climate change and displaying good health in various growing environments.

## 2. Results

### 2.1. The GPFA124 Clone Is More Vigorous and Less Affected by Rust Than the Inbred Line Caturra

**Figure 1:**
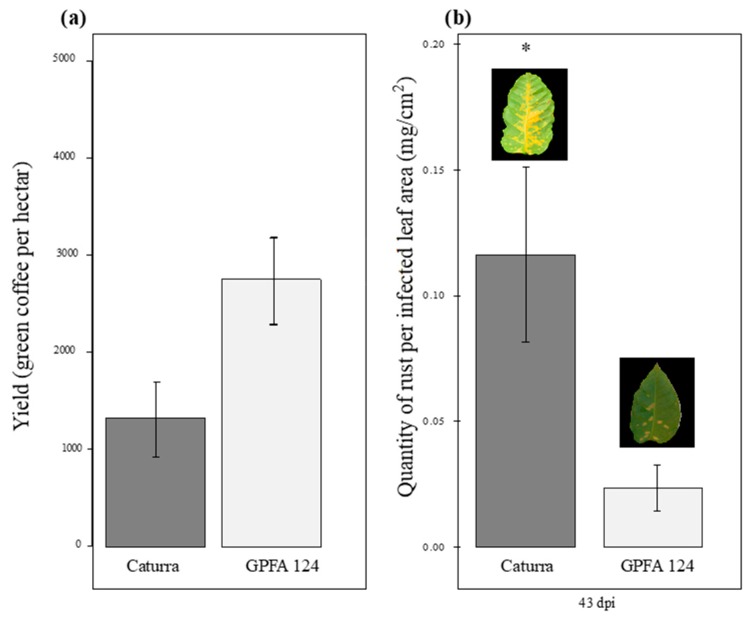
Comparison of agronomic performance in two different genotypes. (**a**) Yield in field conditions. Yield measured as green coffee per hectare results from field evaluations conducted in Equator and Colombia between 2004 and 2009. Caturra line and GPFA124 hybrid are in dark and light gray, respectively. (**b**) Quantity of rust per infected leaf area (also named ‘sporulation’) produced by *H. vastatrix* 43 days post-inoculation in abiotic stress conditions (1000 PAR and low levels of nitrogen fertilization). The inbred line Caturra and the hybrid GPFA124 are, respectively, in dark and light grey. The data are means of the rust weight harvested on the coffee tree 43 days post-inoculation per infected leaf area ± SD (*n* = 4). Means for a genotype followed by an asterisk (*) are significantly different according to student tests (*p* < 0.05). The yield was higher and the sporulation was lower, in the GPFA124 hybrid compared to the inbred Caturra line.

In Ecuador and Colombia, field evaluations conducted between 2004 and 2009 showed average yield values of 1332.13 and 2756.8 kg of green coffee per hectare for Caturra and GPFA124, respectively (Figure 1a). Every year, the yields in GPFA124 fields were double that of Caturra fields. Moreover, less intense rust attacks have been observed in the clone than in the standard Caturra variety. A study conducted in controlled conditions showed similar results. Caturra and GPFA124 were grown under abiotic stress conditions (light intensity of 1000 PAR and low nitrogen fertilization) during 11 weeks prior to inoculation with *Hemileia vastatrix*. The latent period lasted 21 days for Caturra versus 37 days for the clone. The penetration or colonization of host tissues was strongly limited in the hybrids. The quantity of rust produced per infected leaf area, 43 days post-inoculation, was five times lower in the GPFA124 clone than in the standard Caturra variety (Figure 1b).

### 2.2. GPFA124 Clone Has a Better Performance Index Resulting from an Increase in Photosynthetic Electron Transport Chain Efficiency 

**Figure 2:**
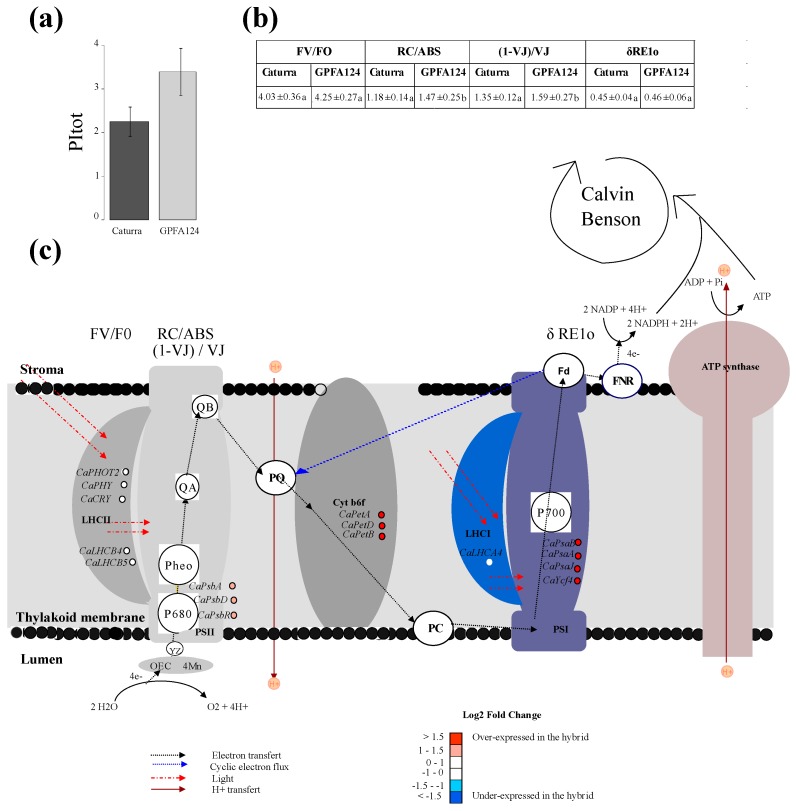
Study of total performance index in two different *C. arabica* genotypes. (**a**) Total performance index (PItotal) at Zeitgeber time 3 (ZT3). Caturra and GPFA124 are in dark and light gray, respectively. (**b**) Table of fluorescence parameters contributing to the total performance index. Data are means ± SD (*n* = 12). Different letters indicate significant differences between the two genotypes according to Mann–Whitney–Wilcoxon’s test (*p* < 0.05). (**c**) Differential expression of electron transfer chain genes at ZT0 in the GPFA124 hybrid compared to Caturra. Incoming light is represented by the dotted red line. Electron transport pathways are shown by black dotted lines. Blue dotted lines indicate cyclic electron flux. Proton fluxes are indicated by continuous red lines. Linear electron flow (LEF) transport is initiated by the simultaneous absorption of light by two antenna complexes (LHCII and LHCI). The absorbed energy is then transferred to the reaction center chlorophylls P680 and P700. The excited state, P680*, donates an electron to pheophytin which, in turn, reduces the primary PQ electron acceptor, QA. QA reduces the secondary PQ electron acceptor, QB. On the donor side of PSII, the hole remaining in P680+ is filled by a redox-active tyrosine, YZ, which subsequently reduces OEC. Electrons are then transferred from PSII through the PQ pool to Cytb6f, which pumps two additional protons across the membrane for each oxidized plastoquinol. Electrons are transported from Cytb6f to PSI by the soluble electron carrier plastocyanin (PC). PSI acts as light-driven plastocyanin FD oxidoreductase. Ultimately, FNR reduces NADP^+^ to NADPH at the expense of reduced FD. Cyclic electron flow (CEF) proceeds through PSI and Cytb6f. Electron transport is coupled to proton translocation into the thylakoid lumen, and the resulting pH gradient drives ATP synthase to produce ATP. The reducing power generated via the so-called light reactions, NADPH, as well as the energy available from ATP hydrolysis, are critical for producing sugars from CO_2_ through the Calvin–Benson cycle. OEC, oxygen-evolving complex; YZ, redox-active tyrosine; PSII, photosystem II; Pheo, pheophytin; QA, primary electron acceptor quinone molecule; QB, secondary electron acceptor quinone molecule; PQ, plastoquinone; Cytb6f, cytochrome b6f; PC, plastocyanin; LHCI, light-harvesting complex I; PSI, photosystem I; FD, ferredoxin; FNR, ferredoxin–NADP+ reductase. The names of the different parameters contributing to PItot (FV/T0; RC/ABS; (1 − VJ)/VJ; δRE1o) are written above the process they measure. Levels of differential expression at ZT0 between the inbred Caturra line and GPFA124 hybrid are represented with colored dots. Red and blue colors represent significant up- and downregulation, respectively, in the GPFA124 hybrid compared to the inbred Caturra line (corrected Bonferroni *p*-value < 0.05). The genes represented are phototropin 2 (CaPHOT2; Cc02_g31790), phytochrome (CaPHY; Cc02_g36930), cryptochrome (CaCRY; Cc10_g07160; Cc03_g05480), light-harvesting complex II chlorophyll a/b binding protein 4 (CaLHCB4; Cc06_g01460), light-harvesting complex II chlorophyll a/b binding protein 5 (CaLHCB5; Cc10_g16210), photosystem II 10 kDa polypeptide (CaPsbR; Cc05_g15930), photosystem II protein D1 (CaPsbA, gene1), photosystem II protein D2 (CaPsbD, gene28), cytochrome f (CaPetA, gene57), cytochrome b6/f complex subunit IV (CaPetD, gene77), cytochrome b6 (CaPetB, gene76), photosystem I P700 apoprotein A1 (CaPsaA, gene36), photosystem I P700 apoprotein A2 (CaPsaB, gene35), photosystem I subunit IX (CaPsaJ, gene66), photosystem I assembly protein Ycf4 (CaYcf4, gene55) (Table S1).

In the GPFA124 clone, a net increase in the efficiency of photosynthetic light-dependent reactions was observed, in comparison to Caturra, through an evaluation of the total performance index (PItotal) for energy conservation from photons absorbed by PSII antenna until the reduction of PSI acceptors (Figure 2a). Changes in this potential for the photochemical utilization of light absorbed in PSII antennae can provide insight into changes in plant photosynthetic performance. It is a multi-parametric expression of four independent parameters: F_v_/F_0_, RC/ABS, (1 − V_J_)/V_J_ and δRE1o (2b). F_v_/F_0_ represents the performance due to the trapping probability, and RC/ABS represents the contribution of the density of active reaction center (in the sense of quinine acceptor (Q_A_) reducing) on a chlorophyll basis. (1 − V_J_)/V_J_ represents the performance due to the conversion of excitation energy to photosynthetic electron transport, in other words, it is the probability that a trapped electron is transferred. δRE1o represents the efficiency of the electron transport flux from QB until the PSI end electron acceptors. Clearly, the highest PItotal value found in the GPFA124 clone is attributable to an increase in RC/ABS and (1 − V_J_)/V_J_. No significant differences have been observed between Caturra and the clone in the energy trapping probability (F_v_/F_0_) and in the efficiency of the electron transport flux from QB until the PSI end electron acceptors (δRE1o) (Figure 2b). The increase in the latter parameters in the clone reflects the upregulation of PSII reaction centers. Indeed, the expression of genes encoding PSII reaction center core proteins D1 and D2 (CaPsbA and CaPsbD) was more than two times higher in the clone than in Caturra. CaPsbR associated with the oxygen-evolving complex of photosystem II was also significantly overexpressed (Figure 2c). Genes encoding light-harvesting complex proteins (CaPHOT2, CaPHY, CaCRY, CaLHCB1, CaLHCB4, and CaLHCB5) showed the same level of expression in both genotypes, explaining the equivalent trapping efficiency in the clone compared to Caturra. However, δRE1o had similar values in both genotypes, even if cytochrome b6f and PSI genes were overexpressed in the clone (Figure 2c). This could be explained by the fact that 1 − FI/FM, which is assumed to provide insight into the cyclic electron flux, was significantly higher in GPFA124 (*p* = 0.05, student test). In the case of PSI cyclic electron transfer, an electron is transferred from Fd to plastoquinone (PQ) and, from there, the electrons are transferred back to PSI. Cyclic electron transfer mediates the transfer of protons from stroma to lumen. It can limit photooxidative stress by favoring the ATP to NADPH output ratio.

### 2.3. Higher Chlorophyll Content in the GPFA124 Clone Is Explained by Biosynthetic and Catabolic Pathway Alteration

**Figure 3:**
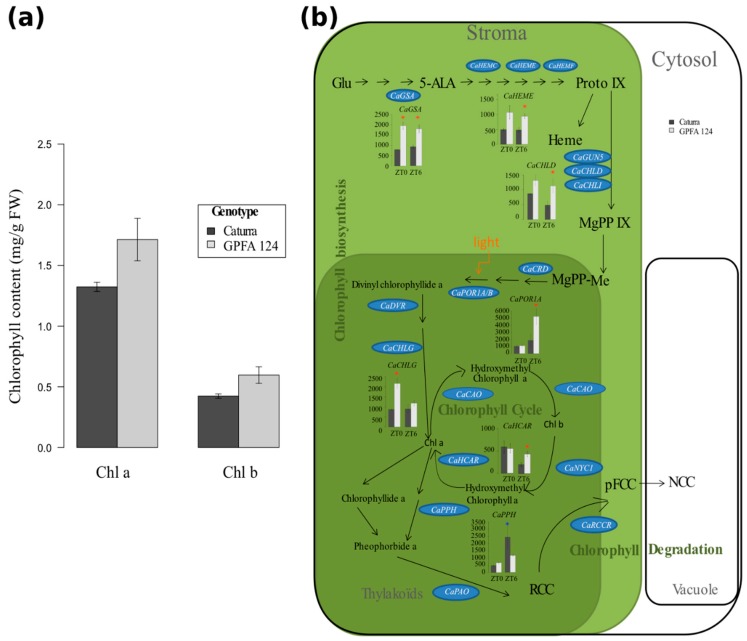
Regulation of the chlorophyll biosynthetic and catabolic pathways in two different *C. arabica* genotypes. (**a**) Increase in chlorophyll (Chl; a,b) content in the GPFA124 hybrid at ZT6. Data are means ± SD (*n* = 6) and were collected during two independent experiments in March 2016 and March 2017. Caturra and GPFA124 are in dark and light gray, respectively. (**b**) Changes in expression of genes involved in the biosynthesis and degradation of chlorophyll in the GPFA124 hybrid and inbred Caturra line at ZT0 and ZT6. Genes found without ambiguity in *Coffea canephora* and *Coffea arabica* genomes were written on the picture in blue ovals. When differences in gene expression between Caturra and GPFA124 are significant at ZT0 or ZT6 (corrected Bonferroni *p*-value < 0.05), relative log expression (rle) normalized data are plotted at ZT0 and ZT6. Significant differential expressions are displayed with red and blue asterisks when the GPFA124 hybrid showed higher and lower gene expression, respectively (deseq2, corrected Bonferroni *p*-value < 0.05). Genes encode the following enzymes: glutamate-1-semialdehyde 2,1-aminotransferase (CaGSA; Cc10_g12640), porphobilinogen deaminase (CaHEMC; Cc05_g16090), uroporphyrinogen II decarboxylase (CaHEME; Cc05_g11120), coproporphyrinogen III oxidase (CaHEMF; Cc09_g04450), Mg-chelatase(CaGUN5;Cc06_g17100, CaCHLD; Cc01_g06000,CaCHLI; Cc07_g18500), Mg-protoporphyrin IX monomethylester cyclase (CaCRD; Cc06_g22740), protochlorophyllide oxidoreductase (CaPOR1A; Cc05_g12370, CaPOR1B; Cc05_g06850), divinyl chlorophyllide a 8-vinyl-reductase (CaDVR; Cc03_g02320), chlorophyll synthase (CaCHLG; Cc06_g01120), chlorophyllide a oxygenase (CaCAO; Cc10_g11980), chlorophyll b reductase (CaNYC1; Cc06_g09730), hydroxymethyl chlorophyll a reductase (CaHCAR; Cc03_g07000), pheophytinase (CaPPH; Cc04_g14700), pheophorbide a oxygenase (CaPaO; Cc01_g10220), red chlorophyll catabolite reductase (CaRCCR; Cc03_g11370). Glu, hlutamic acid; 5-ALA, 5-aminolevulinic acid; proto IX, protoporphyrinogen IX; MgPP IX, Mg-protoporphyrin IX; MgPP-Me; Mg-protoporphyrin IX monomethyl ester; chla, chlorophyll a; chlb, chlorophyll b; RCC, red chlorophyll catabolite; PFCC, fluorescent chlorophyll catabolite; NCC, non-fluorescent chlorophyll catabolite (Table S2).

The total chlorophyll content in the clone was 32% higher than in Caturra (Figure 3a). Chlorophyll b increased slightly more than chlorophyll a. GFPA plants accumulated 41% more chlorophyll b and 30% more chlorophyll a. The higher chlorophyll content in the GPFA124 clone originated from an increased expression of genes encoding enzymes associated with chlorophyll biosynthesis and a reduced expression of genes encoding enzymes associated with chlorophyll degradation (Figure 3b). The genes related to chlorophyll biosynthesis are split in two co-expression clusters: Cluster 3 contains, among others, carbon metabolism-related genes, and Cluster 5 also contains CaPHOT2 coding for light-harvesting protein. Certain genes encoding enzymes involved in chlorophyll biosynthesis (CaGSA, CaHEME, CaCHLD, CaPOR1A, and CaCHLG) were significantly overexpressed in GPFA124 at ZT0 and/or at ZT6 (Figure 3b). CaPOR1A gene encoding protochlorophyllide oxidoreductases mediates the only light-requiring step in chlorophyll biosynthesis in higher plants. CaPOR1A expression was threefold higher in the GPFA124 hybrid than in Caturra at ZT6 (a similar result was obtained by qRT-PCR analysis). CaHCAR encoding the hydroxymethyl chlorophyll a reductase, an enzyme involved in the chlorophyll cycle, was 2.3 times overexpressed in the GPFA124 clone. The expression of CaPPH encoding the pheophytinase, an enzyme involved in chlorophyll catabolism, was 2.12 times underexpressed in the GPFA124 clone (Figure 3b).

### 2.4. Photosynthetic Carbon Metabolism Is Altered in the GPFA124 Clone

**Figure 4:**
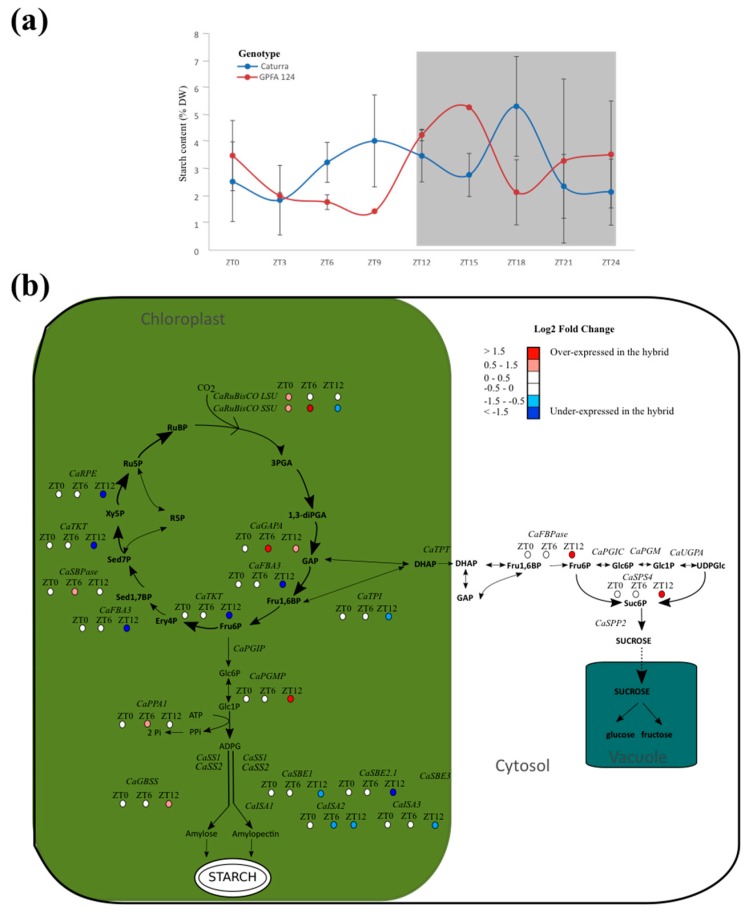
Altered photosynthetic carbon metabolism in F1 hybrid. (**a**) Starch circadian cycle. Data are means ± SD (*n* = 3). Caturra and GPFA124 are in blue and red respectively. A grey background indicates dark period. (**b**) Altered expression of genes involved in photosynthesis carbon metabolism at ZT0, ZT6, and ZT12. Genes found without ambiguity in *Coffea canephora* and *Coffea arabica* genomes were written on the picture. When differences in gene expression between Caturra and GPFA124 are significant at ZT0, ZT6, or ZT12 (corrected Bonferroni *p*-value < 0.05), log2 fold change value is indicated by colored dot. Red, white, and blue colors indicate overexpression, equivalent expression, and underexpression in the GPFA124 hybrid compared to Caturra. Genes encodes the following enzymes: RuBisCo (CaRuBisCO LSU; gene52, CaRuBisCO SSU; Cc00_g15710), NADP-glyceraldehyde phosphate dehydrogenase (CaGAPA; Cc11_g00610), plastidic triose phosphate isomerase (CaTPI; Cc02_g16530), plastidic aldolase (CaFBA3; Cc11_g09330), transketolase (CaTKT; Cc02_g11220), sedoheptulose-1,7-bisphosphatase (CaSBPase; Cc02_g06960), ribulose phosphate epimerase (CaRPE; Cc04_g15130, triose phosphate translocator (CaTPT; Cc02_g13860), cytosolic fructose-1,6-bisphosphatase (CaFBPase; Cc10_g14740), cytosolic phosphoglucoisomerase (CaPGIC; Cc10_g02960), cytosolic phosphoglucomutase (CaPGM; Cc11_g08200), UDP-glucose pyrophosphorylase (CaUGPA; Cc07_g12830), sucrose phosphate synthase (CaSPS4; Cc10_g16060), sucrose 6-phosphate phosphatase (CaSPP2; Cc06_g06940), plastidic phosphoglucose isomerase (CaPGIP; Cc10_g15670), plastidic phosphoglucomutase (CaPGMP; Cc02_g03610), inorganic pyrophosphatase (CaPPA1; Cc02_g20460), soluble starch synthase (CaSS1; Cc02_g01480, CaSS2; Cc07_g03430), granule-bound starch synthase (CaGBSS; Cc08_g16970), starch branching enzyme (CaSBE1; Cc10_g00640, CaSBE2.1; Cc06_g05400, CaSBE3; Cc05_g14070), isoamylase (CaISA1; Cc10_g11550, CaISA2; Cc08_g00630, CaISA3; Cc10_g06640). For simplicity, only the main pathway of carbon is shown, and the use of dihydroxyacetone (DHAP) as a substrate in the two transketolase reactions and of glyceraldehyde-3-phosphate (GAP) as a substrate in the second aldolase reaction leading to the formation of sedoheptulose-1,7-bisphosphate (Sed1,7BP) have been omitted (Tables S3 and S4).

Starch content followed a circadian rhythm for both genotypes and showed one peak during the night at ZT15 for GPFA124, and two peaks at ZT9 and ZT18 for Caturra (Figure 4a). Even if the transitory starch content accumulation over 24 hours was similar for both genotypes (27.7% and 27.2% for Caturra and GPFA124, respectively), the rhythm was different. The peak of starch for the GPFA124 hybrid occurred between those of Caturra. At ZT0, the starch content of the clone is 1.5 times higher. Between ZT3 and ZT9, when photosynthesis is at its most important, Caturra produced and accumulated starch while the clone consumed its reserves. The maximal starch content in leaves is similar for both genotypes (5% DW), but the timing is different. The maximum is reached at ZT15 and ZT18 for the clone and Caturra, respectively.

An overexpression of genes encoding RuBisCO large and small subunits was observed in the hybrid at the beginning of the day (Figure 4b). Genes involved in the CO_2_ acceptor RuBP were underexpressed in the hybrid at the end of the day, whereas the sucrose and starch biosynthesis pathways are enhanced. CaGAPA and CaSBPase are highly correlated (r2 = 0.94, *p*-value = 2.42 × 10^−8^). Indeed, glyceraldehyde-3-phosphate (GAP) produced by GAPA is used as a substrate in the second aldolase reaction, leading to the formation of sedoheptulose-1,7-biphosphate (Sed1,7BP), which is SBPase substrate. This could explain the overexpression of CaSBPase at ZT12. For clarity, this pathway has not been drawn in the Figure. At dusk, genes involved in amylopectin biosynthesis (CaSBE1, CaSBE2 CaISA2, and CaISA3) are underexpressed in the GPFA124 clone.

### 2.5. Metabolic Processes Begin Sooner and Are More Stable in GPFA124 Clone

Enrichment in gene ontology analysis showed that metabolic processes begin sooner in the hybrid. Indeed, more significant GO terms were found at ZT0 on upregulated genes in the hybrid (Table S5). At ZT0, upregulated genes in the inbred line are mainly related to transcription, initiation, and RNA metabolic processes, whereas upregulated genes in the hybrid are mainly related to the generation of precursor metabolites and energy, photosynthesis, the heterocycle metabolic process, and ion transmembrane transport. At ZT6, upregulated genes in the inbred line are mainly related to the carbohydrate metabolic process, protein polymerization, and the cell wall and lipid metabolism process, whereas upregulated genes in the hybrid are mainly related to translation, electron carrier activity, photosynthesis, the macromolecule biosynthetic process, and homeostasis. At ZT12, genes upregulated in the inbred line are mainly related to translation and cellular biosynthetic processes whereas no significant GO terms were found within upregulated genes in the hybrid. Enrichment in the cell wall metabolic process term occurred only in genes upregulated in the inbred line, whereas enrichment in the cellular homeostasis term occurred only in genes upregulated in the hybrid.

### 2.6. The Higher Vigor in Clone GPFA124 May Be Explained by an Alteration of Different Pathways Linked with the Primary Photosynthetic Metabolism

**Figure 5:**
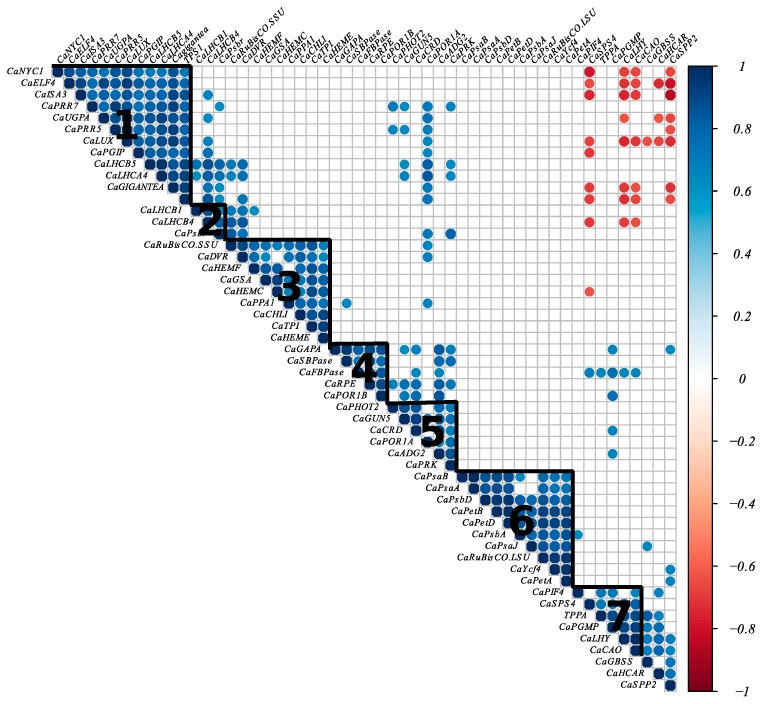
Heat map of circadian clock, photosynthesis, and chlorophyll metabolism gene correlations. A heat map of correlations for 54 genes involved in the circadian clock, photosynthesis, and chlorophyll metabolism was created following hierarchical clustering based on the Pearson correlation coefficient. Colors denote Pearson correlation coefficient between genes. Significant positive and negative correlations are in blue and red, respectively (*p* < 0.001). We identified seven clusters, annotated from 1 to 7 in the heat map.

We performed hierarchical clustering based on the Pearson correlation coefficient which revealed seven clusters of genes (Figure 5). Some genes involved in photosynthesis, chlorophyll, and carbon metabolism formed clusters with genes involved in the circadian clock. For example, the first cluster contained genes involved in the circadian clock, genes encoding light-harvesting complex proteins (CaLHCB5 and CaLHCA4), and genes involved in starch and sucrose biosynthesis, and CaNYC, which is involved in the chlorophyll cycle. Cluster 7 included CaLHY and CaPIF4 belonging to the circadian clock genes, CaCAO implicated in chlorophyll cycle, and CaSPS4, CaTPPA, and CaPGMP involved in sucrose, trehalose, and starch biosynthesis, respectively. The second cluster consists of genes encoding for the light-harvesting complex and PSII proteins. Cluster 3 contains mainly genes involved in chlorophyll biosynthesis and the nuclear gene encoding the small subunit of RuBisCo. Cluster 4 included genes involved in photosynthetic carbon metabolism and CaPOR1B implicated in chlorophyll biosynthesis. Cluster 5 is composed of CaPHOT2 encoding light-harvesting complex protein, genes implicated in chlorophyll biosynthesis, and Calvin cycle genes. Cluster 6 contains chloroplast genes of the photosynthetic electron transport chain and the chloroplast gene encoding the large subunit of the RuBisCo.

### 2.7. The Higher Vigor in the Hybrid May Be Explained by an Alteration of the Circadian Clock

**Figure 6:**
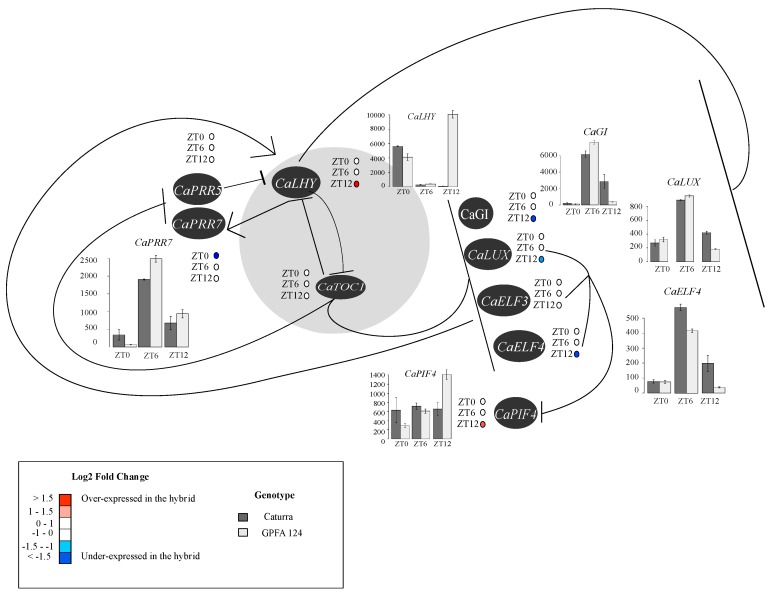
Altered expression patterns of circadian clock genes in the F1 hybrid during the day. The core clock is represented in gray. Arrows represent activation and horizontal line repression. Levels of differential expression at ZT0, ZT6, and ZT12 between the inbred Caturra line and GPFA124 hybrid are represented with colored dots. Red and blue colors represent significant up- and downregulation, respectively, in the GPFA124 hybrid compared to the inbred Caturra line (corrected Bonferroni *p*-value < 0.05). When differences in gene expression between Caturra and GPFA124 are significant at ZT0, ZT6, or ZT12 (corrected Bonferroni *p*-value < 0.05), relative log expression (rle) normalized data are plotted at ZT0, ZT6, and ZT12. Caturra and GPFA124 are in dark and light gray, respectively.

RNAseq analysis of circadian clock genes performed during daytime showed alterations in the GPFA124 clone compared to the Caturra line. CaGIGANTEA, CaLUX-ARRYTHMO, and CaELF4 are negatively correlated with CaLHY. CaLHY was expressed 0.9-fold lower at ZT0 and 15.27-fold higher at dusk (ZT12) in the hybrid. CaPRR7 was also repressed in the clone at dawn. Therefore, the strong CaLHY overexpression in GPFA124 clone at ZT12 is correlated to a downregulation of CaGIGANTEA, CaLUX-ARRYTHMO, and CaELF4, and an overexpression of CaPIF4 (Figure 6). Regarding CaTOC1, no significant differential expression has been observed regardless of the method of analysis used. Most of the photosystem, chlorophyll biosynthesis and catabolism, and photosynthetic carbon metabolism genes, contain circadian-associated 1 binding site (CBS), G-box, morning element (ME), evening element (EE), and LUX binding site (LBS), and are potentially under the control of CaLHY and the Evening complex (Tables S6 and S7).

The genes represented are pseudo-response regulator 5 (CaPRR5; Cc02_g00820), pseudo-response regulator 7 (CaPRR7; Cc06_g03460), late elongated hypocotyl (CaLHY; Cc02_g39990), timing of cab expression 1 (CaTOC1; Cc04_g14990), GIGANTEA (CaGi; Cc10_g15270), LUX-ARRYTHMO (CaLUX-ARRYTHMO; Cc06_g20160), early flowering 3 (CaELF3; Cc08_g10110), early flowering 4 (CaELF4; Cc04_g01390), and phytochrome-interacting factor 4 (CaPIF4; Cc05_g00300). In the GPFA124 hybrid, at dusk, upregulation of CaLHY leads to downregulation of CaGI, CaLUX, and CaELF4, and upregulation of CaPIF4 (Table S8).

## 3. Discussion

Growth in plants is regulated by environmental factors, as well as by carbon availability, developmental stage and, also, the intrinsic performance of genotypes. The interaction between these factors determines the phase of maximal growth rate in different plant organs [27]. In our experiment, environmental factors, carbon availability, and developmental stage are similar for both the GPFA124 clone and Caturra. We have shown that the GPFA124 clone, which is phenotypically superior to Caturra for production, was also superior for rust incidence. In this study, we try to understand the reasons of this greater vigor.

Accurate control of carbon partitioning, starch degradation, and sucrose export rates is crucial to avoid carbon starvation, ensuring optimal growth whatever the photoperiod [28]. Since higher chlorophyll content is not direct proof of better photosynthetic efficiency, we compared the efficiency of photosynthetic light-dependent reactions by measuring chlorophyll *a* fluorescence in the hybrid and Caturra. Chlorophyll *a* fluorescence is a very sensitive probe of the physiological status of leaves and plant performance in a wide range of situations [29]. Here, we have shown that the combination of chlorophyll *a* fluorescence measurement and global transcriptional activity in leaf cells provides a powerful method to analyze photosynthesis efficiency. RNAseq analysis performed during daytime showed alterations in many genes involved in primary photosynthetic metabolism in the GPFA124 clone compared to the Caturra line.

In the GPFA124 clone, the net increase in photosynthesis light-dependent reaction efficiency was due to a higher density of active reaction center (correlated with higher chlorophyll content), and to a better conversion of excitation energy to photosynthetic electron transport. The increase in these parameters in the hybrid reflects the upregulation of PSII reaction centers. In a coherent way, the genes involved in these mechanisms are overexpressed in the clone. The overexpression of PSI genes, combined with an increased Handy PEA 1-FI/FM indicator, shows an increase in cyclic electron transfer and a better ATP and NADPH output ratio. The measure of chlorophyll fluorescence showed the superiority of the clone in terms of electron transport chain efficiency and, probably, lower ROS production.

In photosynthetic cells, starch is synthesized mostly using a fraction of the CO2-fixed carbon and temporarily stored in the chloroplasts. This starch is called ‘transitory’, as it is synthesized and degraded within a 24 h window [30]. The adjustment in the rate of starch degradation during the night is dictated by the anticipation of dawn, as determined by the circadian clock [31]. Control of the supply of carbohydrate for growth during the night is a major and previously overlooked function of circadian mechanisms in plants [32].

At dawn, the level of starch is 1.5× higher in the hybrid leaves. Moreover, genes coding for RuBisCo subunits were overexpressed in the hybrid at dawn, leading to better CO_2_ assimilation. Indeed, the large RuBisCo subunit gene belongs to the cluster of co-expression of chloroplast genes. At dusk, we observed an enhanced carbon reduction in sucrose and starch, and reduced CO_2_ acceptor regeneration in the hybrid. It suggested that the GPFA124 clone has already accumulated all reserves, whereas the inbred Caturra line still needed to assimilate carbon through the Calvin cycle. Gene ontology enrichment analysis showed that metabolic processes occurred later in the inbred line and it is as if the hybrid wakes up early to promote photosynthesis and biomass accumulation. At dusk, the starch biosynthesis pathway is enhanced in favor of amylose biosynthesis, whereas genes involved in amylopectin biosynthesis are underexpressed in the GPFA124 hybrid. Amylopectin accounts for 80% of starch. The underexpression at ZT12 of CaSBE1, CaSBE2, CaISA2, and CaISA3 could explain the earlier peak in starch content in the clone. The rate of starch degradation is tightly regulated so that energy stores are not depleted before photosynthesis can resume [31]. Starch accumulation, by supplying an outlet for photosynthetic end products when exports fail to evacuate them effectively, makes it possible to inhibit downregulation of photosynthesis. Thus, starch accumulation is an indicator of poor sugar-export efficiency [33,34,35]. In Caturra, the continuous increase in starch content between ZT3 and ZT9 indicated low sugar-export efficiency. Since the clone consumes starch and displays more efficient photosynthesis, sugar-export efficiency and sugar available for metabolism must be considerably higher compared to Caturra.

In photosynthetic sucrose synthesis, CFBPase catalyzes the first irreversible reaction from fructose-1,6-biphosphate to fructose-6-phosphate, and plays an important regulatory role in sucrose biosynthesis. Cho et al. (2012) [36] showed by generating transgenic *Arabidopsis* plants which overexpressed both TPT and FBPase, that their simultaneous overexpression leads to enhanced growth and increased CO_2_ assimilation rates. In our data, both genes were overexpressed in the GPFA124 clone at ZT12, even if CaTPT overexpression was not significant at 0.05 thresholds. Moreover, sucrose-phosphate synthase SPS4 was overexpressed in the hybrid at ZT12, suggesting an increased photosynthetic capacity. The expression of a SPS gene from maize resulted in increased photosynthetic rates in transgenic tomato plants under light- and CO_2_-saturated conditions [37].

Recent studies on *Arabidopsis* hybrids [17], rice hybrids [21], and maize hybrids [19] clearly demonstrated increased photosynthetic and metabolic activities linked to an altered expression of circadian clock regulators. As well as acting within the clock mechanism, many genes of the circadian core clock regulate sets of other genes (30–40% of *Arabidopsis* genes) that contribute to a wide range of phenotypic traits [38,39]. Considering that the GPFA124 clone is, in fact, a vigorous Arabica genotype F1 hybrid [40], we investigated if a similar relationship between growth vigor and circadian clock could explain its better agronomic performance. As demonstrated in *Arabidopsis* [17,18], we found that CaLHY is repressed at dawn and upregulated at dusk in the GPFA124 clone, compared to Caturra. Since LHY serves as both a transcriptional repressor and activator of target genes, including those in the core circadian oscillator and output pathways, we postulated a similar role of LHY deregulation in coffee growth vigor as observed in *Arabidospis*. The first link between GPFA124 vigor and deregulation of the circadian clock could be explained by the downregulation of evening complex genes. The upregulation of CaLHY at dusk led to a downregulation of CaELF4 and CaLUX-ARRYTHMO, leading to an upregulation of CaPIF4. Since PIF4 codes for a constitutive transcriptional activator promoting hypocotyl growth by targeting gibberellic acids, ethylene, cytokinins, and auxin-associated genes [41,42,43,44,45], the upregulation of CaPIF4 in the GPFA124 hybrid at dusk could explain higher growth. Moreover, GPFA124 showed higher yields. It has been demonstrated that the circadian clock controls key agricultural traits in crop plants. Alterations in the circadian oscillator also have the potential to increase agricultural yields [46]. In soybean, Preuss et al. (2012) [47] observed an increase in seed weight, number of flowers, pods, and nodes by manipulating the abundance of transcripts encoding soybean LHY and TOC1.

CaLHY is an activator for morning-phased central clock genes (PRR7 and PRR9) and photosynthetic genes, including CAB genes. Integrated analysis of transcriptomics and metabolomics revealed that PRR9, 7, and 5, and LHY negatively regulate the biosynthetic pathways of chlorophyll, highlighting them as additional outputs of pseudo-response regulators [48,49], LHY also negatively regulates downstream genes containing evening element (EE) or CCA1 binding sites (CBS) in their promoter [17,18]. Most of the photosystem, chlorophyll biosynthesis and catabolism, and photosynthetic carbon metabolism genes contain CBS, G-box, ME, EE, and LBS, and are potentially under the control of the circadian core clock genes (Tables S1 and S2). Therefore, CaCAO involved in the chlorophyll cycle was highly correlated with CaLHY (*r* = 0.99). Comparative expression analysis of genes involved in chlorophyll metabolism demonstrated that genes involved in chlorophyll biosynthesis are upregulated and genes encoding enzymes associated with chlorophyll degradation are downregulated in the GPFA124 clone. Downregulation of CaLHY at dawn could explain the higher chlorophyll content in this clone.

Our transcriptomic approach allowed us to go further in understanding molecular mechanisms. and confirmed that part of the higher performance of the GPFA124 clone can be related to better photosynthesis performance and carbon partitioning. Together, these data offer new insights into a better understanding of the complexity of growth vigor in the coffee tree and are of direct relevance to coffee crop production and for further breeding programs.

## 4. Materials and Methods

### 4.1. Plant Material and Growth Conditions

Two genotypes were studied: one inbred line, *C. arabica* var. Caturra, and one Arabica clone, GPFA124. The Caturra seeds came from La Cumplida research center (Matagalpa, Nicaragua). The intraspecific Arabica clone GPFA124 was vegetatively propagated by somatic embryogenesis at the Nestlé R&D laboratory (Tours, France). The GPFA124 clone was selected for high cup quality, high productivity, and excellent growth behavior in Ecuador and Colombia. The plants were cultivated in a glasshouse under natural daylight (65–75% humidity, 12 h day/12 h night) at IRD (Montpellier, France) in 3 L pots containing a GO M2 (Jiffygroup) potting soil mixture, and watered as necessary.

Leaf samples were collected for RNA extraction and chemical analyses at Zeitgeber time (ZT) point ZT0 (sunrise), ZT3, ZT6, ZT9, ZT12 (sunset), ZT15, ZT18, ZT21, and ZT24. For RNAseq extraction, leaves were snap-frozen in liquid nitrogen and stored at −80 °C until RNA extraction. For chemical analyses, leaves were sampled, flash frozen in liquid nitrogen, and then freeze-dried. During sampling operations, three biological replicates were performed for chemical analysis and RNAseq.

### 4.2. Starch Extraction

The starch content of 30 mg of freeze-dried powder was determined using the total starch kit GOPOD (d-glucose, K-Gluc, Megazyme International, Bray, Co Wicklow, Ireland). After elimination of soluble sugars and of the soluble products of starch degradation, the residue was successively hydrolyzed into glucose units with α-amylase and amyloglucosidase. The resulting d-glucose was then degraded with glucose oxidase, and the resulting hydrogen peroxide quantified by spectrophotometry at 510 nm after a last enzymatic reaction. Results are determined in triplicate and expressed as percentage of dry weight (DW).

### 4.3. Chlorophyll Extraction

Chlorophyll extraction was performed at ZT6. Leaves were ground in liquid nitrogen in a ball mill (Tissue Lyser, Qiagen, Hilden, Germany) and extraction was carried out by stirring 60 mg of leaves in 1.5 mL of acetone 80% (*v*/*v*) for 4 h at 4 °C in darkness. After centrifugation at 100,000 *g* and 4 °C for 10 min, the supernatant was diluted ten times in acetone 80% (*v*/*v*). The chlorophyll content was calculated using spectrophotometric absorbance (A) at light wavelengths of 645 and 663 nm, and 80% acetone as a control. Chlorophyll content was expressed as milligram of chlorophyll per gram of fresh leaves using the formula: Chlorophyll a (mg g^−1^) = 12.7 × A663 − 2.69 × A645; Chlorophyll b (mg g^−1^) = 22.9 × A645 − 4.86 × A663; Chlorophyll a + b (mg g^−1^) = 8.02 × A663 + 20.20 × A645 [43].

### 4.4. Chlorophyll a Fluorescence

Chlorophyll *a* fluorescence measurements were conducted at ZT3 with a Handy PEA chlorophyll fluorimeter (Handy-Plant Efficiency Analyser, Hansatech Instruments, Norfolk, UK) on mature leaves. Five fluorescence measurements per plant were performed. Leaves were dark-adapted for 20 min prior to measurement. When leaves kept in the dark are illuminated, chlorophyll *a* fluorescence intensity shows characteristic changes called fluorescence transient [50]. Chlorophyll *a* fluorescence transients were induced by 1 s illumination with an array of six light-emitting diodes providing a maximum light intensity of 3000 PAR. The fast fluorescence kinetics (from F_0_ to F_M_, where F_0_ and F_M_ are, respectively, the minimum and maximum measured chlorophyll fluorescence of PSII in the dark-adapted state) were recorded from 10 µs to 1 s. Dark adaptation allowed the PSII electron acceptor pool to be gradually re-oxidized to a point where all PSII reaction centers were capable of photochemistry. Fluorescent transients were analyzed using the JIP test developed by Strasser and Strasser (1995). The JIP test evaluates the balance between total energy inflows and outflows and provides the probable distribution of light energy absorption (ABS) between the events: trapping (TR), electron transport (ET), and dissipation (DI) [51]. We analyzed, in more detail, the performance index of Strasser [52]: PI_total_, total performance index. PI_total_ reflects the potential for energy conservation from exciton to the reduction flux of PSI end acceptors. It is a multi-parametric expression that combines four parameters related to photosynthetic activity: (i) RC/ABS representing the density of active reaction (in the sense of quinine acceptor (QA) reducing) centers on an equal chlorophyll (absorption) basis; (ii) Fv/F0 representing the performance of the light reactions for primary photochemistry, i.e., the performance due to trapping probability; (iii) (1 − VJ)/VJ representing the performance of the dark reactions, i.e., the performance due to the conversion of excitation energy into photosynthetic electron transport; (iv) δRE1o representing the efficiency with which an electron can move from the reduced intersystem electron acceptors to the PSI end electron acceptors [52].

### 4.5. RNA Extraction, RNA Sequencing, and Bioinformatics Analysis

RNA was extracted from 100 mg of leaves as described by [53].

Only samples taken at ZT0, ZT6, and ZT12 were sequenced. RNA sequencing (RNAseq) was carried out by the MGX platform (Montpellier GenomiX, Institut de Génomique Fonctionnelle, Montpellier; http://www.mgx.cnrs.fr/). RNAseq libraries were constructed as described by [54]. Clustering and 100 nt single read sequencing were performed according to the manufacturer’s instructions.

Image analyses and base calling were performed using the HiSeq Control Software and Real-Time Analysis component (Illumina). Data quality was assessed using fastqc from the Babraham Institute (http://www.bioinformatics.babraham.ac.uk/projects/fastqc/) and the Illumina software SAV (Sequence Analysis Viewer, San Diego, CA, USA). We obtained an average of 21 million single end reads per sample.

The 100 nt single reads were aligned to the *C. canephora* reference sequence for genomic sequences [55] because the Arabica sequence was not yet available, and to chloroplast genome sequence of *Coffea arabica* L. [56] using Tophat v2.1.1 [57], with the following parameters: –read-mismatches 3 –read-gap-length 3 –read-edit-dist 3. Unmapped and multi-position matched reads were excluded from analyses, while uniquely mapped reads were counted for each gene model using Htseqcount for subsequent differential expression analysis by DESEQ2 [58]. On average, 75.4% of sequenced reads were mapped per sample to the reference genome.

### 4.6. Validation of RNAseq by qRT-PCR Analysis

In RNAseq studies, qRT-PCR is usually used for validation. Based on bibliographic data, we targeted three key genes of the circadian clock, CcLHY (Cc02_g39990), CcGIGANTEA (Cc10_g15270) and CcLUX-ARRYTHMO (Cc06_g20160), two genes involved in chlorophyll biosynthesis, CcPOR1A (Cc05_g12370) and CcPOR1B (Cc05_g06850), and two genes involved in starch degradation, CcGWD1 (Cc11_g15490), and CcISA3 (Cc10_g06640). PCR experiments were performed as previously described by [59]. Primers were designed using Primer3Plus web-based software (http://www.bioinformatics.nl/cgi-bin/primer3plus/primer3plus.cgi). The specificity of the PCR products generated for each set of primers was verified by analyzing the *Tm* (dissociation) of amplified products. PCR efficiency (*E*) was estimated using absolute fluorescence data captured during the exponential phase of amplification of each reaction with the equation (1 + *E*) = 10^(−1/slope)^ [60]. Expression levels were calculated by applying the formula (1 + *E*)^−ΔΔ*C*_t_^ where Δ*C*_t_, target = *C*_t_, target gene − *C*_t_ CaGAPDH, gene and ΔΔ*C*_t_ = Δ*C*_t_ target − Δ*C*_t_, references sample, with the T0 sample being used as references for each construct. Expression levels were normalized with the expression of the CaGAPDH gene (GB accession number GW445811) as the endogenous control [61]. Gene expression was measured at ZT0, ZT6, and ZT12. The relative log expression normalized data estimated from RNAseq had high correlation with that from the qRT-PCR (*r* = 0.795; Table S9).

### 4.7. Differential Expression Analysis

Before differential expression (DE) analysis, genes whose sum of counts (by summing the counts by repetitions (3) and genotype (2)) was below 45 were discarded. Reads were then standardized across libraries using the normalization procedure in DESEQ2 [58]. Comparison between the inbred Caturra line and the GPFA124 hybrid was performed at ZT0, ZT6, and ZT12. Differential expression was considered at *p* < 0.05.

All genes of interest were compared using BLASTP against The Arabidopsis Information Resource database (TAIR, www.arabidopsis.org) with an *e*-value cut-off of 1 × 10^−5^. We also performed a BLASTP search against the *C. arabica* phytozome database with e value cut-offs of 1 × 10^−15^.

### 4.8. Statistics

All statistical analyzes were performed using R 3.2.4 software. A Student’s test was performed to test the effect of genotype on the quantity of rust per infected leaf area at 43 dpi, handy pea parameters, and metabolites (stats package). Tests for normality and equality of variance (Shapiro test and Levene’s test, car package) were performed. Alternatively, the non-parametric Mann–Whitney–Wilcoxon test was conducted (stats package).

A Fisher’s exact test (*p* < 0.05) was used to determine if the circadian genes within the differential expressed gene set were present in greater numbers than expected by chance. A heat map was generated using the corrplot package in R. Genes were ordered following hierarchical clustering on the Pearson correlation coefficient using the Ward method. Gene Ontology (GO) analysis was performed using singular enrichment analysis in agriGO V2.0 [62].

### 4.9. Assessment for Rust Resistance

The inbred Caturra line and the GPFA124 hybrid were cultivated in a phytotron (65%–75% humidity, 12 h day/12 h night, day/night temperature: 27 °C/22 °C) at CIRAD (Montpellier, France) [29]. These lines were not expected to carry any specific genetic resistance to prevalent rust races. Water was supplied every day, and they were fertilized with 39 mg of MS/2 medium, 3 mg of KCl, and 35 mg of NH_4_NO_3_ once a week for 11 weeks, i.e., a total of 134 mg of nitrogen. They received a photosynthetically active radiation (PAR) of 1000 µmol∙m^−2^s^−1^ mimicking the light intensity perceived in a full sun system. Rust infestation and macroscopic monitoring was described in [29].

## Supplementary Materials

Supplementary materials can be found at http://www.mdpi.com/1422-0067/20/3/736/s1.

## Abbreviations

CBS	CCA1 binding sites
CCA1	circadian clock-associated
DW	dry weight
GO	gene ontology
